# A type 1 diabetes genetic risk score discriminates between type 1 diabetes and type 2 diabetes in a Chinese population

**DOI:** 10.1007/s00125-025-06455-x

**Published:** 2025-06-26

**Authors:** Jingyi Hu, Guozhi Jiang, Jiabi Qin, Shuoming Luo, Baoqi Fan, Zhiguo Xie, Raymond Wan, Xia Li, Claudia H. T. Tam, Zhenqian Wang, Jin Ding, Ying Xia, Yuanqin Yang, Jian Lin, Gechang Yu, Ping Jin, Cadmon K. P. Lim, Hong Kong Diabetes Biobank Study Group, Andrea O. Y. Luk, Wing Yee So, Juliana C. N. Chan, Congyi Wang, Jiaqi Huang, Michael N. Weedon, William A. Hagopian, Richard A. Oram, Ronald C. W. Ma, Yang Xiao, Zhiguang Zhou

**Affiliations:** 1 National Clinical Research Center for Metabolic Diseases, Key Laboratory of Diabetes Immunology, Ministry of Education, and Department of Metabolism and Endocrinology, The Second Xiangya Hospital of Central South University, Changsha, China; 2 School of Public Health (Shenzhen), Sun Yat-sen University, Shenzhen, China; 3 Department of Epidemiology and Health Statistics, Xiangya School of Public Health, Central South University, Changsha, China; 4 National Health Committee Key Laboratory of Birth Defect for Research and Prevention, Hunan Provincial Maternal and Child Healthcare Hospital, Changsha, China; 5 Department of Medicine and Therapeutics, The Chinese University of Hong Kong, Hong Kong, China; 6 Hong Kong Institute of Diabetes and Obesity, The Chinese University of Hong Kong, Hong Kong, China; 7 CUHK-SJTU Joint Research Center in Diabetes Genomics and Precision Medicine, The Chinese University of Hong Kong, Hong Kong, China; 8 Laboratory for Molecular Epidemiology in Diabetes, Li Ka Shing Institute of Health Sciences, The Chinese University of Hong Kong, Hong Kong, China; 9 Department of Endocrinology, The Third Xiangya Hospital of Central South University, Changsha, China; 10 Li Ka Shing Institute of Health Sciences, The Chinese University of Hong Kong, Hong Kong, China; 11 Department of Respiratory and Critical Care Medicine, the Center for Biomedical Research, NHC Key Laboratory of Respiratory Diseases, Tongji Hospital, Tongji Medical College, Huazhong University of Sciences and Technology, Wuhan, China; 12 Shanxi Bethune Hospital, Shanxi Academy of Medical Science, Tongji Shanxi Hospital, Third Hospital of Shanxi Medical University, the Key Laboratory of Endocrine and Metabolic Diseases of Shanxi Province, Taiyuan, China; 13 Department of Clinical and Biomedical Sciences, University of Exeter, Exeter, UK; 14 Department of Paediatrics, Indiana University School of Medicine, Indianapolis, IN USA; 15 Department of Medicine, University of Washington, Seattle, WA USA; 16 Institute of Biomedical and Clinical Science, University of Exeter Medical School, and The Academic Kidney Unit, Royal Devon and Exeter NHS Foundation Trust, Exeter, UK

**Keywords:** Clinical diabetes, Clinical science, Genetics / Epidemiology (all), Genetics of type 1 diabetes, Prediction and prevention of type 1 diabetes

## Abstract

**Aims/hypothesis:**

We aimed to generate a population-specific type 1 diabetes genetic risk score (GRS) and assess whether it could improve discrimination between type 1 diabetes and type 2 diabetes in a Chinese population.

**Methods:**

We performed a genome-wide association analysis on 1303 individuals with type 1 diabetes and 2236 control individuals. An independent replication cohort of 501 individuals with type 1 diabetes and 853 control individuals was used to validate the top common variant associations. HLA typing data were used to identify tag SNPs for *DQA1-DQB1* haplotypes. We integrated significant signals to construct a Chinese type 1 diabetes GRS (C-GRS). The accuracy of the C-GRS was tested in an independent validation cohort consisting of 262 individuals with type 1 diabetes, 1080 individuals with type 2 diabetes and 208 control individuals.

**Results:**

We identified a variant, rs10232170, in *BMPER* as a possible novel type 1 diabetes locus (*p*=9.897×10^−9^). We identified tag SNPs for 13 *DQA1-DQB1* haplotypes and 12 non-*DQA1-DQB1* loci. Integrating 33 significant SNPs from HLA and non-HLA regions, C-GRS demonstrated high discriminative power for type 1 diabetes (AUC=0.876). It was tested in an independent validation cohort and showed high discrimination (AUC 0.871 for type 1 diabetes vs control group, 0.869 for type 1 diabetes vs type 2 diabetes). The C-GRS outperformed a European-derived GRS (0.871 vs 0.773, and 0.869 vs 0.793, respectively).

**Conclusions/interpretation:**

A type 1 diabetes C-GRS comprising 33 SNPs was highly discriminative of type 1 diabetes risk in the Chinese population and could aid in discriminating between type 1 diabetes and type 2 diabetes. This study highlights the potential of genetic information in improving prediction and precision diagnosis of type 1 diabetes in the Chinese population.

**Data availability:**

The raw sequencing data and summary statistics of genomic DNA derived from human samples have been deposited at the China National Center for Bioinformation (https://ngdc.cncb.ac.cn/omix) under accession number PRJCA023730.

**Graphical Abstract:**

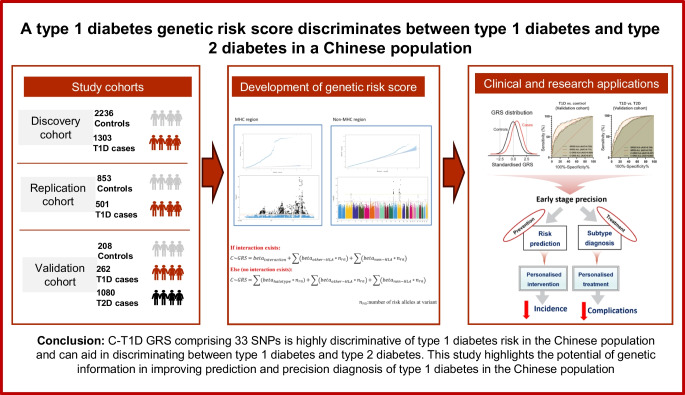

**Supplementary Information:**

The online version contains peer-reviewed but unedited supplementary material available at 10.1007/s00125-025-06455-x.


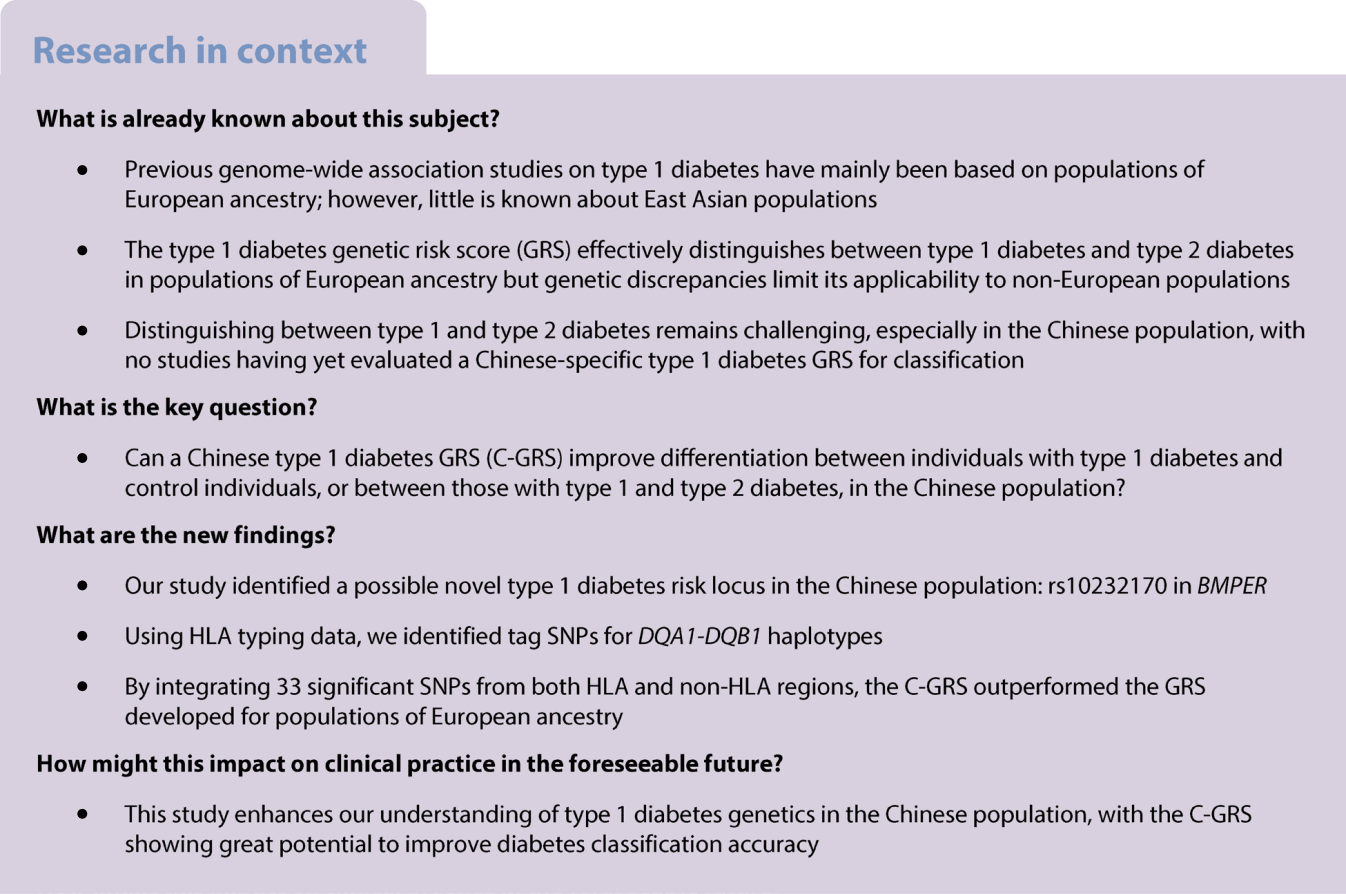


## Introduction

Type 1 diabetes results from an autoimmune attack on pancreatic beta cells, culminating in a profound lack of insulin [1]. At present, methods to differentiate type 1 diabetes from type 2 diabetes involve clinical phenotypes such as age of onset, BMI and biomarkers such as C-peptide levels and islet autoantibodies. Increasing rates of obesity in type 1 diabetes, the presence of ketosis-prone type 2 diabetes and idiopathic type 1 diabetes, and limited autoantibody testing in some regions make it an increasingly difficult challenge to accurately classify type 1 diabetes and type 2 diabetes. Approximately 40% of adults with type 1 diabetes are initially misdiagnosed as having type 2 diabetes [2, 3]. This challenge is particularly pronounced in the Chinese population, where young-onset type 2 diabetes at lower BMI and older age at type 1 diabetes diagnosis are common [4–6]. Misdiagnosing type 1 diabetes as type 2 diabetes often leads to delayed insulin treatment, which can lead to severe complications [7, 8]. Therefore, it is crucial to search for new methods to discriminate the different types of diabetes to enable precision treatment to prevent complications.

Given the distinct genetic backgrounds of type 1 diabetes and type 2 diabetes, a genetic risk score (GRS) offers promise as a tool for accurate classification [9, 10]. A type 1 diabetes GRS, comprising 30 type 1 diabetes-associated SNPs, can be a useful tool to aid the discrimination between type 1 diabetes and type 2 diabetes in populations of European ancestry [9, 11]. GRS2, which improved the SNP capture of HLA *DR-DQ* risk, further improved discriminative power for the classification of diabetes type [12–14]. The above two GRS models were derived from populations of European ancestry and their discriminatory power may differ in cohorts of different ancestries due to discrepancies in genomic architecture and allele frequency [11, 12, 15]. Indeed, the genetic background of type 1 diabetes in the Chinese population significantly differs from that in the populations of European ancestry in the most important genetic regions for type 1 diabetes, namely the HLA *DR-DQ* haplotypes [16, 17]. The first Chinese type 1 diabetes genome-wide association studies (GWASs) identified three novel loci [18] but SNPs tagging HLA *DR-DQ* haplotypes have not been reported or validated in Chinese cases. Additionally, no studies have assessed the utility of a Chinese-optimised type 1 diabetes GRS for diabetes classification.

In this study we used GWAS to identify genetic variants associated with type 1 diabetes in Chinese. Additionally, we aimed to identify, for the first time, accurate SNP tags for HLA *DR-DQ* haplotypes in the Chinese population to quantify type 1 diabetes risk associated with specific haplotypes and their interactions. Using this information, we constructed a Chinese-specific GRS (C-GRS) and evaluated its ability to differentiate between participants with type 1 diabetes and control participants, as well as between participants with type 1 diabetes and those with type 2 diabetes.

## Methods

### Participants

**Fig. 1:**
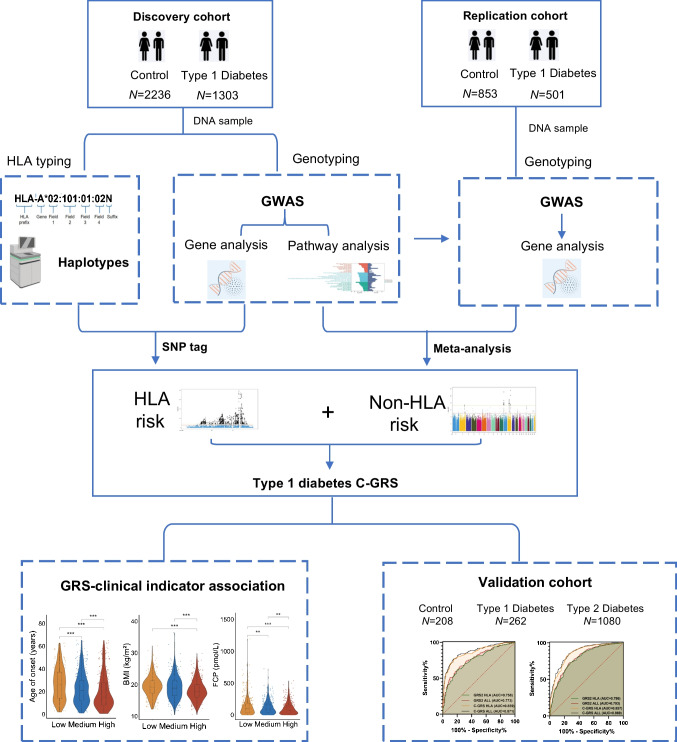
Flow chart of the study design. A two-stage GWAS (including a discovery cohort and an external replication cohort) in 1804 individuals with type 1 diabetes and 3089 control individuals was conducted. By combining HLA typing data, we generated SNP tags for HLA haplotypes in Chinese individuals and used them together with non-HLA loci to construct a C-GRS. We then identified the association between C-GRS and clinical indicators and validated the ability of C-GRS to differentiate between individuals with type 1 diabetes and control individuals, and between individuals with type 1 diabetes and those with type 2 diabetes, in an independent validation cohort

We conducted a two-stage case–control study of individuals with type 1 diabetes and control individuals in the Chinese population (Fig. 1). The discovery cohort included 1303 participants with type 1 diabetes and 2236 control individuals recruited from the Second Xiangya Hospital of Central South University between January 2013 and October 2020, with HLA typing performed on 2342 of the participants. The replication cohort consisted of 501 individuals with type 1 diabetes and 853 control individuals recruited from Hong Kong between 1995 and 2019 [19, 20]. The validation cohort included 262 participants with type 1 diabetes, 1080 with type 2 diabetes (aged >20 and <45 years [21]) and 208 control participants recruited from the Second Xiangya Hospital of Central South University between January 2021 and April 2023. Adult-onset type 1 diabetes was defined as the onset of type 1 diabetes in participants aged ≥20 years [22]. The inclusion and exclusion criteria for participants are shown in electronic supplementary material (ESM) Methods and ESM Fig. 1. Sex or gender was based on self-report by participants. All samples were collected with appropriate assent or informed consent from all participants or their guardians. This study was approved by the Ethics Committee of Second Xiangya Hospital of Central South University (no. 2019-Research-135) and ethics approval for recruitment of the Hong Kong participants was provided by the Joint Chinese University of Hong Kong-New Territories East Cluster Clinical Research Ethics Committee (CREC-2002.183-T, CRE-2013.187 and CRE-2013.304). The studies were conducted in accordance with the principles of the Declaration of Helsinki.

### Genotyping and quality control

**Fig. 2:**
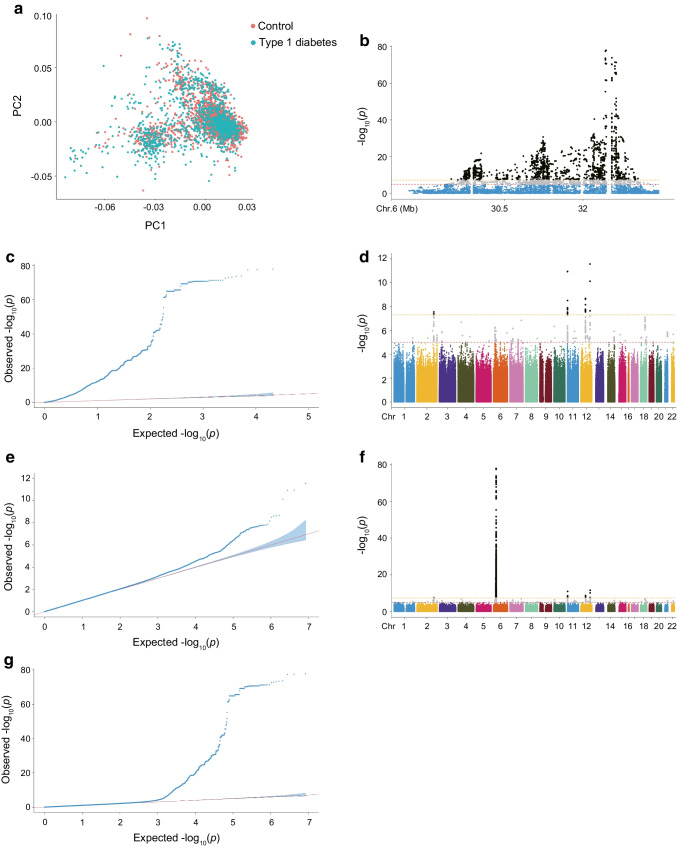
GWAS results in the discovery cohort. (**a**) Principal component analysis revealed the population structure of the control group and the disease group. (**b**, **d**, **f**) Manhattan plots display genome-wide association results for type 1 diabetes in the HLA region (**b**), non-HLA region (**d**) and across all autosomes (**f**). (**c**, **e**, **g**) Quantile–quantile plots illustrate associations with type 1 diabetes risk in the HLA region (**c**), non-HLA region (**e**) and across all autosomes (**g**). Observed *p* values are plotted as a function of theoretical *p* values. The blue areas represent the 95% CI derived from a null distribution of *p* values. PC, principal component

Genotyping was performed using the Illumina Asian Screening Array (ASA). For sample quality control, we excluded individuals with ambiguous sex, overall call rate <95%, extreme heterozygosity or any duplicate or related individuals (PI_HAT ≥0.2). For SNP quality control we excluded SNPs that were monomorphic, had a minor allele frequency (MAF) of <0.05, had a Hardy–Weinberg equilibrium of *p*<1×10^−4^ or had a missingness rate of >0.05. Finally, 484,700 qualified SNPs from 1275 type 1 diabetes cases and 2100 controls were included in the analysis. Principal component analysis showed that the cases and controls in the discovery cohort were genetically matched (Fig. 2a). The genomic inflation factor (λ) of 1.021 indicated that there was little evidence of systematic bias between the participants with type 1 diabetes and control participants. Genotyping of the Hong Kong participants was performed using the Illumina Omni2.5+ exome array and the Illumina ASA.

### Imputation

The HLA and non-HLA regions were imputed using different strategies. To impute the HLA region, we used the SNP2HLA v1.0.3 (https://software.broadinstitute.org/mpg/snp2hla/) and a previously published reference dataset of Chinese Han individuals, which included classical typing for HLA types A, B and C, and *HLA-DRB1*, *HLA-DQA1*, *HLA-DQB1*, *HLA-DPA1* and *HLA-DPB1* [23]. To impute the non-HLA region, we employed the IMPUTE2 software V.2.2.2 (https://mathgen.stats.ox.ac.uk/impute/impute_v2.html), which automatically identifies the best-matching haplotypes from the entire population of the 1000 Genomes Project. To enhance imputation performance, we also implemented a phased strategy using the SHAPEIT software Version 2 (https://mathgen.stats.ox.ac.uk/genetics_software/shapeit/shapeit.html). Imputed variants meeting a minimum MAF threshold of 0.5% and imputation information score greater than 0.4 were retained. In total, we imputed 150 alleles at the following loci: *HLA-A/B/C/DRB1/DQA1/DQB1/DPB1*. Alleles were imputed to a resolution of four digits, resulting in an average imputation *r*^2^ of 0.970.

### HLA *DQA1-DQB1* haplotypes

Using the alleles from imputed data, we inferred the most likely *DQA1-DQB1* haplotype combinations for each individual, based on the haplotypes in the Chinese populations. For haplotypes present in more than 0.5% of case and control individuals, we assessed them as predictive factors for type 1 diabetes within a regression model and calculated whether there were interactions among these haplotypes. We used HLA typing data to identify variants that would best mark these haplotypes from their correlation *r*^2^ and *D′* statistics.

### Interaction modelling

A total of 15 possible *DR-DQ* haplotype pairings (haplogenotypes) were identified. To assess whether interactions existed between them, we generated multiplicative interaction terms from genotype dose values. Using each haplogenotype as a covariate and interaction terms as the independent variable, we applied logistic regression to assess the strength of the interactions.

### Additional loci

Given the long-range linkage between HLA class I, class II and class III loci, we performed logistic regression for 106 imputed alleles from HLA loci outside of *DR-DQ*, adjusting for previously identified *DR-DQ* haplotypes, using a Bonferroni-corrected *p* value threshold (*p*<4.72×10^−4^).

### Generating the GRS

The GRS was computed by integrating risks associated with the *HLA-DQ* region, in addition to risk from non-*HLA-DQ*, and the non-HLA gene region [13]. For haplotypes within the *DR-DQ* region that had already been identified with interactions, each individual was assigned a score based on these interactions. For haplotypes without identified interactions, non-HLA-DQ alleles, and SNPs in non-HLA regions (*p*<5×10^−8^), the score was calculated as the sum of each allele multiplied by its weighted beta coefficient. For each allele, we took the natural logarithm of the OR to generate the β statistic. Thus, a positive β value indicated that the allele was associated with increased risk, while a negative value indicated a protective effect.

### Statistical analyses

To identify and validate associations, we used Plink v1.9 (https://www.cog-genomics.org/plink/1.9/) [24] to treat the participants with type 1 diabetes and control individuals as logical values of 1 and 0, respectively, and established the logistic regression on the SNPs. When estimating ORs and 95% CIs, sex and principal components with *p* values <0.05 were included as covariates. Linkage disequilibrium (LD) score regression was used to assess the genetic correlation between European ancestry and Chinese ancestry individuals [25]. The GWAS data for the Japanese cohort were collected from the GWAS Catalog (https://www.ebi.ac.uk/gwas/studies/GCST90018705) [26]. The methodology for constructing the GRS was referenced from prior publications [13]. To assess model discrimination, the area under the receiver operating characteristic (ROC) curve and area under the precision–recall curve (PRAUC) were calculated. Cox regression analysis was applied to assess the relationship between GRS and age of diagnosis. Differential analysis between GRS and clinical variables was conducted using both parametric and non-parametric tests. The DeLong test was employed to assess differences between ROC curves. Basic statistical analyses were performed using SPSS version 26.0 and R version 4.0.4.

## Results

### GWAS of type 1 diabetes vs control

The study design is illustrated in Fig. 1. The baseline characteristics of the discovery, replication and validation cohorts are shown in ESM Table 1. In the discovery cohort, compared with controls, individuals with type 1 diabetes were younger on average, and 53.6% were male. In the discovery cohort, GWAS of 1303 type 1 diabetes participants and 2236 controls identified significant type 1 diabetes genetic associations both within and outside of the HLA region (Fig. 2). A total of 5817 SNPs reached the threshold of *p*<1.00×10^−5^, including 369 located outside the HLA region. We identified 17 SNPs that showed consistency with previous reports in populations of European ancestry, reaching a significance level of *p*<1×10^−5^ (ESM Table 2). These included the chromosomal regions 2q33.2 (*CTLA4*), 6q21.2 (*HLA*), 11p15.5 (*INS-IGF2*), 12q13.2 (*RAB5B-SUOX-RPS26-ERBB3*), 14q32.2 (*VRK1*), 18p11.21 (*PTPN2*) and 18q22.2 (*CD226*). Using our GWAS data and publicly available GWAS summary statistics from European populations, we found a moderate genetic correlation (*r*_g_=0.41) between European and Chinese ancestries, indicating a certain degree of population heterogeneity.

Subsequently, we performed GWAS validation using an independent replication cohort consisting of 501 participants with type 1 diabetes and 853 control individuals from Hong Kong. Meta-analysis of the top SNPs in the discovery cohort and replication cohort revealed 202 SNPs that reached genome-wide significance (*p*<5.00×10^−8^). In addition to the SNPs within the HLA region, there were eight SNPs in non-HLA regions (Table 1), including chromosomal regions 2q33.2 (*CTLA4*), 7p14.3 (*BMPER*), 10p15.1 (*IL2RA*), 11p15.5 (*INS-IGF2*), and 12q13.2 (*SUOX-RPS26-SH2B3-ATXN2*). We identified a SNP rs10232170 in *BMPER* as a possible novel risk locus. Although it was not replicated in the replication cohort, it reached genome-wide significance in the meta-analysis (*p*=9.897×10⁻^9^; Table 1 and ESM Fig. 2). Additionally, we identified pathways of the validated signals in type 1 diabetes. We found that the pathways of ‘MHC class II receptor activity’ and ‘peptide antigen binding’ were the most enriched biological processes in participants with type 1 diabetes when compared with control participants (ESM Fig. 3). Furthermore, when we compared our results with previously published data from the Japanese population, the most significant susceptibility loci remained in the HLA region (ESM Table 3).

**Table 1:** Genomic regions achieving genome-wide significance validated in replication cohort (*p*<5.00×10^−8^)

Chr	BP	Representative marker	A1	A2	MAF	β	*p* value	Candidate gene	Reference
2q33.2	204729153	rs231770	T	C	0.322	0.273	3.299×10^–8^	*CTLA4*	[9, 13]
6p21.32	32636289	rs9274655	C	T	0.411	1.323	9.822×10^–63^	*MHC*	[9, 13]
7p14.3	34181522	rs10232170	T	C	0.280	–0.391	9.897×10^–9^	*BMPER*	/
10p15.1	6079344	rs11256442	C	T	0.493	–0.245	8.638×10^–9^	*IL2RA*	[13]
11p15.5	2182224	rs689	T	A	0.035	1.031	5.371×10^–12^	*INS-IGF2*	[9, 13]
12q13.2	56394954	rs773125	G	A	0.264	0.301	1.257×10^–8^	*SUOX-RPS26-SH2B3-ATXN2*	[9, 13, 18]

### HLA type 1 diabetes associations

We identified tag SNPs for 13 HLA *DQA1-DQB1* haplotypes that were associated with type 1 diabetes in the discovery cohort (ESM Table 4), following the approach used in a previous study [13]. Interestingly, DQA1*0301-DQB1*0302 (*DQ8*) was not found to be significantly associated with type 1 diabetes in our data, despite its significant association in studies involving populations of European ancestry. After identifying *DQ* haplotypes, we detected and used their interactions to refine the estimation of type 1 diabetes risk. We identified 15 *DQ* haplotypes with significant interaction terms and calculated the OR specific to each interaction (ESM Table 5).

We identified 12 HLA region SNPs that were not representative of *DR-DQ* alleles but each was independently associated with type 1 diabetes (ESM Table 6). Some known type 1 diabetes-associated alleles in the HLA class I or II (except for *DQ*) gene regions, as well as some intergenic loci, were also present. By combining the (13 + 12) total HLA region SNPs, the C-GRS achieved good discrimination in the discovery cohort (ROC AUC=0.864).

### Non-HLA type 1 diabetes associations

We identified eight non-HLA loci, each of which, again, was independently associated with type 1 diabetes (ESM Table 7). Together, these non-HLA SNPs discriminated type 1 diabetes in the discovery cohort (ROC AUC=0.641).

### The C-GRS using 33 SNPs is highly discriminative of type 1 diabetes vs control in Chinese individuals

**Fig. 3:**
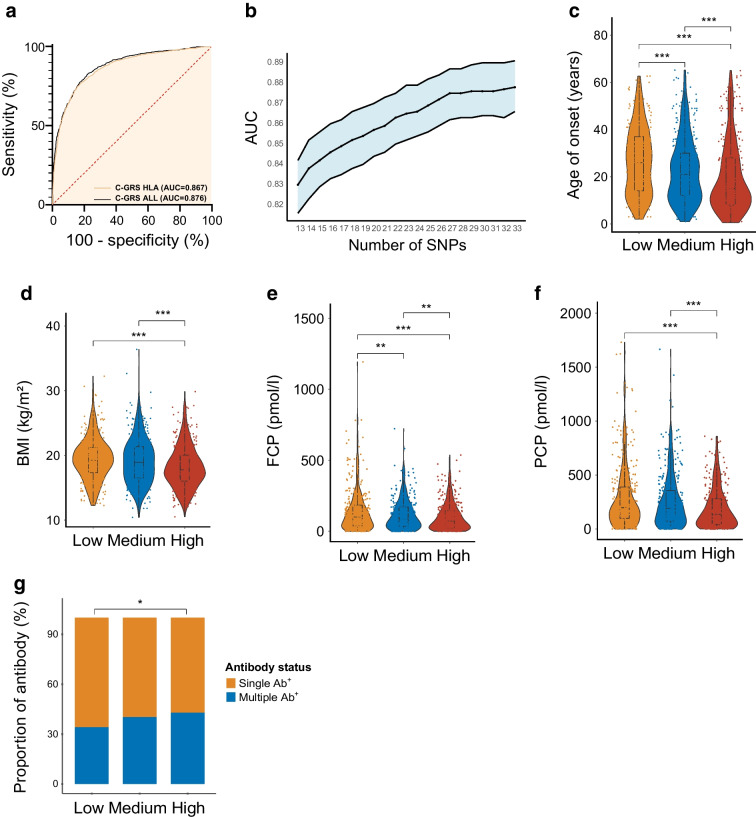
The diagnostic efficacy of C-GRS and its association with clinical indicators. (**a**) AUC results from ROC analysis for C-GRS in the discovery cohort. (**b**) The gradual improvement in the power of the C-GRS as additional SNPs were included. The blue shaded area represents the 95% CI. (**c**–**g**) A higher C-GRS was associated with an earlier age (**c**), lower BMI (**d**), lower fasting (**e**) and postprandial C-peptide levels (**f**) and higher proportion of multiple positive autoantibodies (**g**) at diagnosis. Box plots show median ± quartiles, and the whiskers extend from the hinge to the largest or smallest value no further than 1.5-fold of the IQR. *p<0.05, **p<0.01, ***p<0.001. Ab, autoantibody; FCP, fasting C-peptide; PCP, postprandial C-peptide

**Fig. 4:**
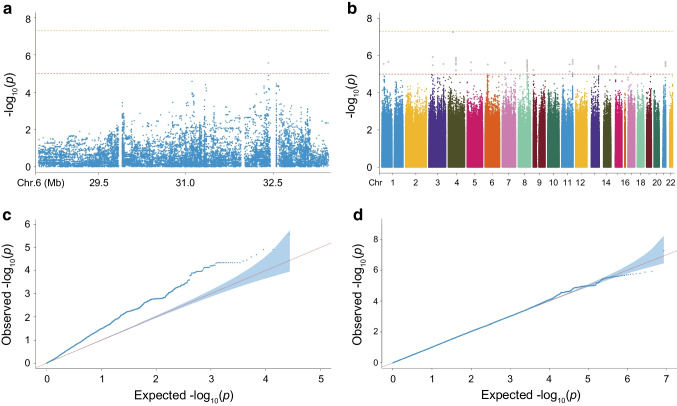
GWAS After adjustment for C-GRS. (**a**, **b**) Manhattan plot showing the genome-wide association results for type 1 diabetes in the HLA region (**a**) and the non-HLA region (**b**). (**c**, **d**) Quantile–quantile plots were used to assess the association between type 1 diabetes risk and the HLA region (**c**) and the non-HLA region (**d**). The observed *p* values are plotted as a function of the expected *p* values. The blue areas represent the 95% CI derived from a null distribution of *p* values

The final combined type 1 diabetes C-GRS used 33 SNPs, including 13 *DR-DQ* haplotypes, 12 other HLA SNPs and eight non-HLA SNPs. This C-GRS showed high discrimination of type 1 diabetes, with ROC AUC=0.876 (Fig. 3) and PRAUC=0.820 (ESM Fig. 4) in the discovery cohort. We found no genome-wide significant SNP associations when including C-GRS as a covariate, indicating good capture of type 1 diabetes-associated information (Fig. 4). The efficiency (or cost) of the C-GRS depends on the SNP count. We sequentially added SNPs outside the *DR-DQ* region, ranking them by *p* value to compare AUC discriminative ability, and found no significant AUC increase from 29 to 30 SNPs (AUC=0.874 vs AUC=0.875, *p*>0.05, Fig. 3b). Subsequently, we stratified the analysis by age of onset and calculated the AUC. We found that C-GRS shows a higher AUC in the youth-onset group (AUC=0.911) compared with the adult-onset group (AUC=0.849), with a significant difference (*p*=3.446×10^−7^, ESM Fig. 5).

**Fig. 5:**
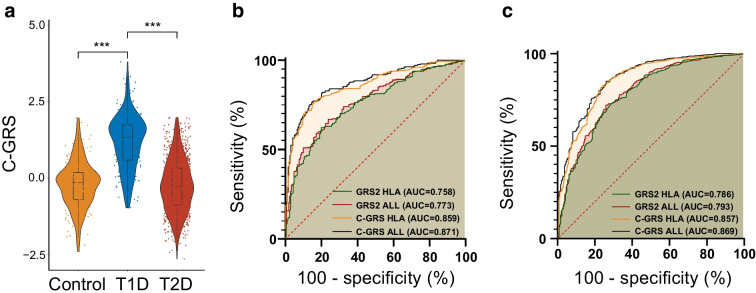
The validation of the discrimination performance of C-GRS in an independent cohort. (**a**) The C-GRS of type 1 diabetes was significantly higher than that of control individuals and those with type 2 diabetes in the validation cohort. (**b**) The type 1 diabetes C-GRS has high discriminatory power in distinguishing individuals with type 1 diabetes from both control individuals. (**c**) C-GRS demonstrated strong discriminatory ability between individuals with type 1 diabetes and those with type 2 diabetes. ***p<0.001. T1D, type 1 diabetes; T2D, type 2 diabetes

Additionally, we validated the C-GRS in the validation cohort. We observed a significant difference in the C-GRS when comparing participants with type 1 diabetes and the control participants (*p*=4.483×10^−81^, Fig. 5a). Notably, using only the SNPs representing the HLA region, we achieved an AUC of 0.859 in discriminating between participants with type 1 diabetes and control participants (Fig. 5b). When using non-HLA region loci alone to distinguish between individuals with type 1 diabetes and control individuals, an AUC of 0.598 was obtained. When using all SNPs for discrimination, the AUC was 0.871 (Fig. 5b).

Next, we evaluated the performance of the previously established GRS2, derived mainly from populations of European ancestry [13], in our cohort (Fig. 5 and ESM Fig. 6). We found that when using the HLA region loci and all loci of GRS2 to distinguish between type 1 diabetes and control individuals separately, the AUCs were 0.758 and 0.773 (Fig. 5b), respectively. Subsequently, we compared the performance of GRS2 with the newly constructed C-GRS to differentiate control individuals from those with type 1 diabetes in the validation cohort. We found that our newly established C-GRS improved the discriminative power for type 1 diabetes vs control compared with GRS2 (*p*=2.657×10^−6^).

### Associations of type 1 diabetes C-GRS with clinical features

We stratified the C-GRS by tertiles in the discovery cohort. Compared with the low C-GRS group, the high C-GRS group showed an earlier age of type 1 diabetes diagnosis (median [IQR]: 26.0 [14.0–37.0] vs 15.0 [8.0–23.0] years, *p*=1.520×10^−11^, Fig. 3c), lower BMI (median [IQR]: 19.2 [17.3–21.2] vs 17.7 [15.8–20.0] kg/m^2^, *p*=4.875×10^−7^), lower levels of fasting C-peptide (median [IQR]: 100.8 [38.4–184.0] vs 79.0 [31.1–157.0] pmol/l, *p*=7.300×10^−5^) and lower levels of 2 h postprandial C-peptide (median [IQR]: 197.5 [98.7–396.2] vs 157.3 [55.9–298.4] pmol/l, *p*=7.640×10^−7^, Fig. 3d–f). Additionally, the proportion of individuals with multiple autoantibody positivity was higher in the high C-GRS group (34.1% vs 42.9%, *p*=4.030×10⁻^2^, Fig. 3g).

### The type 1 diabetes C-GRS discriminates between clinically defined type 1 diabetes and type 2 diabetes

Next, we calculated C-GRS in a validation cohort, which included 262 individuals with type 1 diabetes (median [IQR]: 4.374 [2.358–5.466]) and 1080 individuals with type 2 diabetes (median [IQR]: 0.049 [−1.545 to 1.672]), with strict clinical criteria employed to define each type of diabetes. The C-GRS was highly discriminative of type 1 diabetes from type 2 diabetes (*p*=7.099×10⁻^72^; Fig. 5a) with an AUC of 0.869 (Fig. 5c). When only using HLA region loci alone or non-HLA GRS, the AUC was 0.857 or 0.588. The DeLong test demonstrated that, compared with GRS2, C-GRS exhibited superior performance in distinguishing between type 1 diabetes and type 2 diabetes in the Chinese population (0.869 vs 0.793, *p*=4.003×10^−5^).

We then determined cutoff values for the clinical classification of individuals with type 1 diabetes or type 2 diabetes. ESM Table 8 provides a set of examples of C-GRS cutoffs, sensitivities and specificities. Scores with 95% specificity for type 1 diabetes or type 2 diabetes were identified as being useful for further classification. A C-GRS of >1.211 was indicative of type 1 diabetes, with 95% specificity and 55% sensitivity (i.e. a C-GRS of 1.211 was at the 95th centile of a control population and 45th centile of the type 1 diabetes cohort). A C-GRS of < −0.407 (38th centile of C-GRS in validation cohort) was indicative of type 2 diabetes, with 95% specificity and 45% sensitivity. The 33-SNP C-GRS also provided excellent discrimination between adult-onset type 1 diabetes and type 2 diabetes (AUC=0.818).

## Discussion

In this study, we conducted a GWAS in the Chinese population to investigate type 1 diabetes susceptibility loci unique to the Chinese. Building upon the GWAS results, we developed a type 1 diabetes C-GRS for diabetes diagnosis and classification. Our C-GRS exhibits high efficacy in distinguishing between individuals with type 1 diabetes and control individuals, as well as between those with type 1 diabetes and type 2 diabetes in Chinese, with replication and validation in two independent cohorts from Hong Kong and mainland China.

Our investigation highlights the possibility that the study of increasing numbers of individuals from non-European ancestry cohorts may reveal novel genetic associations of type 1 diabetes [15, 27]. We found both similarities and discrepancies in type 1 diabetes risk loci when comparing Chinese and European populations. We replicated 17 findings from European ancestry studies. We used a larger threshold in the discovery cohort to select SNPs with the aim of verifying more loci and then applied the genome-wide significance threshold in subsequent replication or meta-analysis. We also identified a possible new genome-wide significant locus that failed to replicate, *BMPER* (encoding bone morphogenetic protein [BMP]-binding endothelial regulator [BMPER]) with the SNP rs10232170, which had not been previously found in European-focused analyses [28, 29] or a recent similarly sized GWAS from China [18], possibly due to ethnic or geographical differences. Additionally, previous GWASs in Chinese populations only validated a limited number of loci. BMPER is an extracellular modulator of the BMP pathway. BMP and its receptors are signalling molecules that belong to the TGF-β superfamily, with TGF-β signalling being an important modulator of inflammation. Interestingly, overexpression of *Tgfb1* has been reported to improve autoimmune beta cell injury in a mouse model of type 1 diabetes [30, 31]. Previous studies have indicated that overexpression of BMPER or the delivery of recombinant BMPER protein can alleviate insulin resistance and hyperglycaemia in diabetic mice, suggesting the protective role of BMPER in glucose homeostasis [32], making this a possible therapeutic target.

It is notable that the strongest association signals for type 1 diabetes lay within HLA class II haplotypes. Similar to European studies, common alleles such as *DR3-DQ2.5* (DRB1*03:01-DQA1*05-DQB1*02:01) showed significant effects. We identified HLA alleles associated with risk that were more prevalent in Chinese (*DR4-DQ3* [DRB1*04:05-DQA1*03:03-DQB1*04:01] and *DR9-DQ3* [DRB1*09:01-DQA1*03:02-DQB1*03:03]) [17]. On the other hand, DQA1*03:01-DQB1*03:02, which confers elevated risk in type 1 diabetes in western populations, did not show significant effect in our cohort. This may be due to its common association in Chinese individuals with protective *DRB1* alleles such as DRB1*04:06, which may neutralise the overall risk of the extended haplotype. While these allele variations might underlie the rarity of type 1 diabetes in Chinese population, the C-GRS based on both HLA and non-HLA associated SNPs showed high discriminatory performance for individuals with type 1 diabetes vs control individuals, with a ROC AUC of 0.876.

To date, most of the GRS studies for type 1 diabetes arise from populations of European ancestry without ideal performance of these GRSs in other ethnic groups [11, 12, 33–35]. This highlights the need to validate GRSs in participants of different ancestry with varying allele frequencies as well as participants of the same ancestry living in different geographical regions to account for gene–environment interactions. Due to the lower incidence rate of type 1 diabetes in China, its presentation at an older average age, and the challenges in establishing large-scale, comprehensive type 1 diabetes cohorts, there have been few studies evaluating the performance of type 1 diabetes GRS in China. Moreover, these studies did not undergo robust external validation, nor did they describe HLA associations or provide a list of SNPs, βs and interaction terms to allow others to generate the score. Our study is the first to develop a C-GRS for the classification of type 1 diabetes and type 2 diabetes specifically in East Asian populations. There is increasing recognition of the contribution of type 1 diabetes to the overall burden of diabetes in adults [7]. Even in European cohorts, over 60% of new type 1 diabetes diagnoses occur in participants over 20 years of age [36–38]. In addition, the incidence and/or ascertainment of type 1 diabetes in China is rising, particularly in individuals over 30 years old [39]. Current diagnostic methods based on islet autoantibodies and beta cell function can be costly and inefficient. These biomarkers might also change over time, resulting in misdiagnosis as type 2 diabetes, incorrect treatment and increased morbidity [8, 40]. By incorporating East Asian-specific type 1 diabetes susceptibility haplotypes (e.g. DQA1*03:03-DQB1*04:01) and type 1 diabetes protective haplotypes (e.g. DQA1*01:02-DQB1*05:02) that had not been included in GRS2 [13], the C-GRS outperformed European ancestry-based GRS2 in distinguishing individuals with type 1 diabetes from control individuals and distinguishing between individuals with type 1 diabetes and those with type 2 diabetes in Chinese populations.

The HLA regions play a key role in immune regulation and are linked to various autoimmune diseases, including type 1 diabetes. In this study, we used tagged SNPs to capture not only type 1 diabetes-susceptible HLA *DR-DQ* haplotypes but also other HLA haplotypes and their interactions, likely associated with dysregulation of immunity [23, 41]. This study is the first to identify SNP tags for HLA *DR-DQ* risk in Chinese individuals and assess interactions between them. Additionally, it includes many class I alleles and other susceptibility information, leading to the derivation of the C-GRS. The number of autoantibodies was also higher in the high C-GRS group. Given the increasingly low cost of GWAS and direct SNP genotyping, this cost-effective method will allow widespread application for knowledge discovery.

Limitations of this study include use of a cross-sectional design, which means we could not directly assess the power of the C-GRS to predict future type 1 diabetes. However, a cross-sectional design offers the most efficient way to have a sufficiently large sample size to assess genetic associations. Additionally, since our study was based on a limited sample of the Chinese Han population, the generalisability of our findings to populations with different ethnic backgrounds is limited. For many GRSs, trans-ancestry approaches have facilitated improved and more generalisable scores that can be applied across the globe. Future research will focus on using methods of trans-ancestral GRS development to combine the new Chinese GWAS with larger European GWAS datasets in order to develop optimised ancestry-specific and/or trans-ancestral GRS. In this context, genetic interactions, gene–environment interactions and the background incidence of diabetes in different populations will likely need to be considered. The number of male and female participants was comparable in both the discovery and replication cohorts of this study, and the analysis was adjusted for sex. Therefore, the results are considered applicable to both male and female populations.

In summary, our GWAS has advanced the genetic understanding of type 1 diabetes in the Chinese population. We have successfully established a C-GRS for type 1 diabetes tailored for the Chinese population, and validated its effectiveness as a discriminatory diagnostic tool for different types of diabetes. The superior performance in discriminating between type 1 diabetes and type 2 diabetes using the C-GRS compared with the GRS2 derived from Europeans underscores the necessity of establishing population-specific GRSs or multiethnic GRSs for implementing precision medicine in diabetes.

## Supplementary Information

Below is the link to the electronic supplementary material.

ESM (PDF 671 KB)

## Data Availability

The raw sequencing data and summary statistics of genomic DNA derived from human samples have been deposited at the China National Center for Bioinformation (https://ngdc.cncb.ac.cn/omix) under accession number PRJCA023730.

## Abbreviations

BMP: Bone morphogenetic protein
BMPER: BMP-binding endothelial regulator
C-GRS: Chinese-specific genetic risk score
GRS: Genetic risk score
GWAS: Genome-wide association study
MAF: Minor allele frequency
PRAUC: Area under the precision–recall curve
ROC: Receiver operating characteristic
