# Supplementary material for: A type 1 diabetes genetic risk score discriminates between type 1 diabetes and type 2 diabetes in a Chinese population
Source: Diabetologia. 2025 Jun 26;68(9):1969–82. doi: 10.1007/s00125-025-06455-x (PMC12361272; doi:10.1007/s00125-025-06455-x)

Supplemental Materials

Methods

Participants

Discovery cohort:

All participants with diabetes recruited in this study fulfilled the 1999 World Health Organization criteria for diabetes [1]. The diagnostic criteria of type 1 diabetes were as follows: a. consistent with ADA criteria [2]; b. insulin dependence within 6 months of diagnosis; c. presence of at least one positive autoantibody (GADA, IA-2A, or ZnT8A); d. presence or absence of diabetic ketosis or ketoacidosis at onset. The exclusion criteria included non-Han Chinese individuals with type 1 diabetes, other types of diabetes, and individuals with missing information. The control group was recruited from the general population in the same geographic region, with normal glucose tolerance determined by Oral Glucose Tolerance Test (OGTT), no personal or family history of diabetes, and no history of autoimmune diseases. The exclusion criteria included non-Han Chinese individuals and those with missing information.

Replication cohort [3-4]:

Diagnostic criteria of type 1 diabetes were: a. consistent with ADA criteria [2]; b. insulin dependence within 12 months of diagnosis; c. presence or absence of diabetic ketosis or ketoacidosis at onset. Whilst not all subjects had anti-GAD or anti-IA-2 antibodies measured, the majority had measurements done. Control individuals were recruited from the same geographic area with normal glucose tolerance, as determined by OGTT.

Validation cohort:

All participants with diabetes recruited in this study fulfilled the 1999 World Health Organization criteria for diabetes [1]. The diagnostic criteria of type 1 diabetes were as

follows: a. consistent with ADA criteria [2]; b. insulin dependence within 6 months of diagnosis; c. presence of at least one positive autoantibody (GADA, IA-2A, or ZnT8A); d. presence or absence of diabetic ketosis or ketoacidosis at onset. The exclusion criteria included non-Han Chinese individuals with type 1 diabetes, other types of diabetes, and individuals with missing information. The control group was recruited from the general population in the same geographic region, with normal glucose tolerance determined by OGTT, no personal or family history of diabetes, and no history of autoimmune diseases. The exclusion criteria included non-Han Chinese individuals and those with missing information. The inclusion criteria for participants in the type 2 diabetes group were a diagnosis age between 20 and 45 years, negative GADA test results, treatment with diet, exercise, or oral hypoglycemic agents, or a diagnosis-to-insulin treatment interval of more than one year. Exclusion criteria included non-Han Chinese individuals, known cases of other types of diabetes such as secondary diabetes or gestational diabetes, and individuals with missing information. When validating the GRS in the validation cohort, we excluded samples with missing data in the HLA region or missingness in two or more SNPs.

Our sample size was determined with reference to previous genome-wide association studies on type 1 diabetes and controls [5-6]. For participants aged 18 years or older, informed consent was obtained from the participants themselves. For participants aged <18 years, informed consent was obtained from their legal guardians, and the participants themselves provided verbal or written assent based on their cognitive ability. To compare the genetic risk of type 1 diabetes and type 2 diabetes, standardized z-scores of the GRS were generated for individuals in the validation cohort (Fig. 1 and ESM Table 8).

[1] Alberti KG, Zimmet PZ (1998) Definition, diagnosis and classification of diabetes mellitus and its complications. Part 1: diagnosis and classification of diabetes mellitus provisional report of a WHO consultation. Diabetic medicine: a journal of the British Diabetic Association 15(7): 539-553. 10.1002/(sici)1096-9136(199807)15:7<539::Aid-dia668>3.0.Co;2-s

[2] Association AD (2017) 2. Classification and Diagnosis of Diabetes. Diabetes care 40(Suppl 1): S11-s24. 10.2337/dc17-S005

[3] Luk AO, Lau ES, So WY, et al. (2014) Prospective study on the incidences of cardiovascular-renal complications in Chinese patients with young-onset type 1 and type 2 diabetes. Diabetes care 37(1): 149-157. 10.2337/dc13-1336

[4] Luk AOY, Lau ESH, Lim C, et al. (2019) Diabetes-Related Complications and Mortality in Patients With Young-Onset Latent Autoimmune Diabetes: A 14-Year Analysis of the Prospective Hong Kong Diabetes Register. Diabetes care 42(6): 1042-1050. 10.2337/dc18-1796

[5] Onengut-Gumuscu S, Chen WM, Robertson CC, et al. (2019) Type 1 Diabetes Risk in African-Ancestry Participants and Utility of an Ancestry-Specific Genetic Risk Score. Diabetes care 42(3): 406-415. 10.2337/dc18-1727

[6] Zhu M, Xu K, Chen Y, et al. (2019) Identification of Novel T1D Risk Loci and Their Association With Age and Islet Function at Diagnosis in Autoantibody-Positive T1D Individuals: Based on a Two-Stage Genome-Wide Association Study. Diabetes care 42(8): 1414-1421. 10.2337/dc18-2023

Additional information

<u>Hong Kong Diabetes Biobank Study Group Members</u>

Ronald C.W. Ma [1,2,3,4], Juliana C.N. Chan [1,2,3,4], Risa Ozaki[1,2], Andrea O.Y. Luk[1,2,3,4], Wing-yee So[1,2], Ka-fai Lee[5], Shing-chung Siu[6], Grace Hui[6], Chiu-chi Tsang[7], Kam-piu Lau[8], Jenny Y.Y. Leung[9], Man-wo Tsang[10], Grace Kam[10], Elaine Cheung[10], Ip-tim Lau[11], June K.Y. Li[12], Vincent T.F. Yeung[13], Jo Jo Kwan[13], Samuel Fung[14], Stanley Lo[15], Emmy Lau[15], Yuk-lun Cheng[16], Stephen K.W. Tsui[17], Yu Huang[17], Huiyao Lan[1,3], Weichuan Yu[18], Brian Tomlinson[1], Si Lok[19], Ting-fung Chan[20], Kevin Y.L. Yip[21], Cheuk-chun Szeto[1,3], Xiaodan Fan[22], Nelson L.S. Tang[3, 23], Xiaoyu Tian[17], Claudia H.T. Tam[1,2,4], Guozhi Jiang[1,2,4], Shi Mai[17], Baoqi Fan[1,2,4], Fei Xie[1], Sen Zhang[18], Pu Yu[18], Meng Wang[18], Heung-man Lee[1], Cadmon K.P. Lim [1,2,4], Fangying Xie[1], Alex C.W. Ng[1], Grace P.Y. Cheung[1], Alice P.S. Kong[1,2], Elaine Y.K. Chow[1,2], Ming-wai Yeung[1], Chun-chung Chow[1], Kitty K.T. Cheung[1], Rebecca Y.M. Wong[1], Hon-cheong So[17], Katie K.H. Chan[1,2], Chin-san Law[11], Anthea K.Y. Lock[11], Ingrid K.Y. Tsang[11], Chi-kin Yeung[11], Susanna C.P. Chan[11], Yin-wah Chan[11], Cherry Chiu[2], Chi-sang Hung[10], Cheuk-wah Ho[10], Ivy H.Y. Ng[10], Maria W.H. Mak[7], Kai-man Lee[7], Candy H.S. Leung[13], Ka-wah Lee[12], Hui-ming Chan[12], Winnie Wat[15], Tracy Lau[15], Cheuk-yiu Law[14], Ryan H.Y. Chan[14], Candice Lau[1], Pearl Tsang[1], Vince Chan[1], Lap-ying Ho[1], Eva Wong[1], Josephine Chan[1], Sau-fung Lam[1], Jessy Pang[1], Yee-mui Lee[1]

[1] Department of Medicine and Therapeutics, The Chinese University of Hong Kong, Hong Kong

[2] Hong Kong Institute of Diabetes and Obesity, The Chinese University of Hong Kong, Hong Kong

[3] Li Ka Shing Institute of Health Sciences, The Chinese University of Hong Kong, Hong Kong

[4] Chinese University of Hong Kong-Shanghai Jiao Tong University Joint Research Centre in Diabetes Genomics and Precision Medicine

[5] Department of Medicine and Geriatrics, Kwong Wah Hospital, Hong Kong

[6] Diabetes Centre, Tung Wah Eastern Hospital, Hong Kong

[7] Diabetes and Education Centre, Alice Ho Miu Ling Nethersole Hospital, Hong Kong

[8] North District Hospital, Hong Kong

[9] Department of Medicine and Geriatrics, Ruttonjee Hospital, Hong Kong

[10] Department of Medicine and Geriatrics, United Christian Hospital, Hong Kong

[11] Tseung Kwan O Hospital, Hong Kong

[12] Department of Medicine, Yan Chai Hospital, Hong Kong

[13] Centre for Diabetes Education and Management, Our Lady of Maryknoll Hospital, Hong Kong

[14] Department of Medicine and Geriatrics, Princess Margaret Hospital, Hong Kong

[15] Department of Medicine, Pamela Youde Nethersole Eastern Hospital, Hong Kong

[16] Department of Medicine, Alice Ho Miu Ling Nethersole Hospital, Hong Kong

[17] School of Biomedical Sciences, The Chinese University of Hong Kong

[18] Department of Electronic and Computer Engineering, Hong Kong University of Science and Technology, Hong Kong

[19] The Centre for Applied Genomics, The Hospital for Sick Children, Toronto, Canada

[20] School of Life Sciences, The Chinese University of Hong Kong, Hong Kong

[21] Department of Computer Science and Engineering, The Chinese University of Hong Kong, Hong Kong

[22] Department of Statistics, The Chinese University of Hong Kong, Hong Kong

[23] Department of Chemical Pathology, The Chinese University of Hong Kong, Hong Kong

Tables

ESM Table 1. Demographics and baseline characteristics of discovery cohort, replication cohort and validation cohort.

	**Discovery cohort**		**Replication cohort**		**Validation cohort**		
	**Type 1 Diabetes (n = 1303)**	**Controls (n = 2236)**	**Type 1 Diabetes (n = 501)**	**Controls (n =853)**	**Type 1 Diabetes (n = 262)**	**Type 2 Diabetes (n = 1080)**	**Controls (n=208)**
Sex (male)	698 (53.6%)	1296 (58.0%)	237 (47.3%)	415 (48.7%)	136 (51.7%)	822 (76.1%)	155 (73.81%)
Age (years)	24.78 (14.71)	45.21 (14.86)	36.85 (15.63)	45.16 (11.42)	21.43 (16.46)	39.26 (4.06)	35.75 (9.28)
Age of onset (years)	22.02 (13.24)	/	25.07 (13.93)	/	20.17 (13.63)	37.64 (6.89)	/
BMI (kg/m^2)	18.97 (3.59)	22.67 (4.32)	22.47 (3.68)	23.78 (3.67)	19.32 (4.53)	25.14 (3.67)	/
FCP (pmol/l)	127.48 (145.82)	/	/	/	115.87 (95.29)	603.37 (355.50)	/
PCP (pmol/l)	271.52 (317.54)	/	/	/	278.67 (328.21)	1579.14 (1017.59)	/
GADA (+)	1145/1303 (87.9%)	/	191/379 (50.4%)	/	115/152 (75.66%)	0/1080 (0%)	/
IA-2A (+)	509/1303 (39.1%)	/	86/302 (28.5%)	/	57/97 (58.76%)	0/176 (0%)	/

ZnT8A (+)	294/1303 (22.6%)	/	/	/	54/103 (52.43%)	0/138 (0%)	/

Data are presented as mean (SD), or n (%). BMI, body mass index; FCP, fasting C-peptide; PCP, postprandial C-peptide; GADA, glutamic acid decarboxylase antibody; IA-2A, insulinoma-2-associated autoantibodies; ZnT8A, zinc transporter-8 antibody.

Not all participants in the replication cohort and validation cohort have tested for autoantibodies.

ESM Table 2. Our discovery cohort yielded consistent results with previously reported T1D susceptibility loci ($P < 1.00\times10^{-5}$).

Chr	SNP	BP	Candidate Gene	A1	OR	SE	MAF		p	Ref
							Case	Control		
2	rs3087243	204738919	*CTLA4*	G	0.7185	0.0684	0.1615	0.2114	6.61×10^{-6}	(Oram RA, Diabetes Care. 2016; Sharp SA, Diabetes Care. 2019)
6	rs72848653	29836602	*HCG4P7*	C	1.5625	0.0703	0.2569	0.1812	1.52×10^{-14}	(Sharp SA, Diabetes Care. 2019)
6	rs9269173	32447188	*HLA-DRB9*	T	5.5449	0.1291	0.2013	0.0435	3.52×10^{-42}	(Sharp SA, Diabetes Care. 2019)
6	rs10947332	32677440	*MTCO3P1*	G	0.2737	0.0960	0.0527	0.1688	3.58×10^{-13}	(Sharp SA, Diabetes Care. 2019)
11	rs3842753	2181060	*INS*	T	3.2508	0.1504	0.0150	0.0471	1.22×10^{-11}	(Sharp SA, Diabetes Care. 2019)
11	rs689	2182224	*INS-IGF2*	A	3.2522	0.1508	0.0149	0.0470	1.18×10^{-11}	(Oram RA, Diabetes Care. 2016; Zhu M, Diabetes Care. 2019)
12	rs705699	56384804	*RAB5B*	G	1.4271	0.0599	0.3181	0.2464	2.18×10^{-8}	(Zhu M, Diabetes Care. 2019)
12	rs773125	56394954	*SUOX*	A	1.4655	0.0611	0.3111	0.2356	2.39×10^{-9}	(Zhu M, Diabetes Care. 2019)
12	rs705704	56435412	*RPS26*	G	1.4698	0.0616	0.2909	0.2182	2.67×10^{-8}	(Bradfield JP, PLoS Genet. 2011)
12	rs705705	56435504	*RPS26*	G	1.4707	0.0616	0.2911	0.2183	2.51×10^{-8}	(Onengut-Gumuscu S, Nat Genet 2015)

12	rs11171739	56470625	*ERBB3*	C	0.7059	0.0604	0.3013	0.2333	4.86×10^{-7}	(Barrett JC, Nat Genet 2009; Onengut-Gumuscu S, Nat Genet. 2015)
12	rs4759229	56474480	*ERBB3*	A	0.7196	0.0613	0.2902	0.2274	3.05×10^{-6}	(Oram RA, Diabetes Care. 2016)
12	rs2292239	56482180	*ERBB3*	T	0.7275	0.0615	0.2838	0.2238	8.81×10^{-6}	(Todd JA, Nat Genet. 2007; Barrett JC, Nat Genet. 2009)
14	rs1456988	98488007	*VRK1*	G	0.7901	0.0540	0.4541	0.5129	6.03×10^{-6}	(Onengut-Gumuscu S, Nat Genet. 2015)
18	rs1893217	12809340	*PTPN2*	A	1.3504	0.0681	0.2292	0.1805	4.03×10^{-6}	(Oram RA, Diabetes Care. 2016; Sharp SA, Diabetes Care. 2019)
18	rs1615504	67526644	*CD226*	T	0.7778	0.0557	0.3886	0.3308	7.23×10^{-6}	(Onengut-Gumuscu S, Nat Genet. 2015; Sharp SA, Diabetes Care. 2019)
18	rs763361	67531642	*CD226*	T	0.7787	0.0557	0.3880	0.3305	9.19×10^{-6}	(Barrett JC, Nat Genet. 2009; Onengut-Gumuscu S, Nat Genet. 2015)

T1D, type 1 diabetes; Chr, chromosome; SNP, single nucleotide polymorphism; BP, base pair; OR, odds ratio; SE, standard error; MAF, minor allele frequency.

ESM Table 3. The association results of the genome-wide significant variants in our study and BioBank Japan.

Chr	BP	Representative Marker	A1	A2	Candidate Gene	Cases	MAF	Beta	*p*
2q33.2	204729153	rs231770	T	C	*CTLA4*	CHN	0.322	0.2730	3.30×10^{-8}
						BBJ	0.386	0.0002	9.95×10^{-1}
						Combined		0.1156	3.21×10^{-4}
6p21.32	32636289	rs9274655	C	T	*MHC*	CHN	0.411	1.3230	9.82×10^{-63}
						BBJ	0.429	0.2078	6.59×10^{-7}
						Combined		0.4403	8.81×10^{-33}
7p14.3	34181522	rs10232170	T	C	*BMPER*	CHN	0.280	-0.3910	9.90×10^{-9}
						BBJ	0.111	-0.0459	4.98×10^{-1}
						Combined		-0.2174	6.07×10^{-6}
10p15.1	6079344	rs11256442	C	T	*IL2RA*	CHN	0.493	-0.2450	8.64×10^{-9}
						BBJ	0.419	-0.0336	4.24×10^{-1}
						Combined		-0.1380	4.02×10^{-6}
11p15.5	2182224	rs689	T	A	*INS-IGF2*	CHN	0.035	1.0310	5.37×10^{-12}
						BBJ	0.035	-0.0091	9.36×10^{-1}
						Combined		0.3676	4.41×10^{-5}
12q13.2	56394954	rs773125	G	A	*RPS26-SUOX-SH2B3-ATXN2*	CHN	0.264	0.3010	1.26×10^{-8}
						BBJ	0.201	-0.0177	7.34×10^{-1}
						Combined		0.1395	1.72×10^{-4}
6p21.32	32484520	rs138748427	A	C	*HLA-DRB5*、*HLA-DRB9*	CHN	0.390	0.7441	4.4×10^{-56}
						BBJ	0.492	0.2913	2.93×10^{-9}
						Combined		0.5266	4.99×10^{-54}
7q32.1	127258384	rs3757787	T	C	*SND1-DT, PAX4*	CHN	0.067	0.0407	5.28×10^{-1}
						BBJ	0.088	0.4363	1.20×10^{-8}
						Combined		0.2048	3.23×10^{-5}
11p15.5	2858546	rs2237897	T	C	*KCNQ1*	CHN	0.346	-0.0313	7.18×10^{-1}
						BBJ	0.392	-0.2431	1.58×10^{-8}

	Combined	-0.2014	1.73×10^{-7}

Chr, chromosome; BP, base pair; MAF, minor allele frequency; CHN, Chinese cohort; BBJ, BioBank Japan.

ESM Table 4. Thirteen HLA-DQ haplotypes were included in the C-GRS.

Marker	Haplotype	r^2	Freq	A1	OR	Beta
rs3134978	*DQA1*05:01-DQB1*02:01*	0.97	0.120	T	10.38	2.34
rs117876414	*DQA1*03:03-DQB1*04:01*	0.91	0.090	A	2.72	1.00
rs9405035	*DQA1*03:0X-DQB1*03:03*	0.80	0.280	A	2.25	0.81
rs79972380	*DQA1*01-DQB1*0602*	0.69	0.030	A	0.09	-2.36
rs201768714	*DQA1*05:05-DQB1*03:01*	0.78	0.060	A	0.37	-0.99
rs35476892	*DQA1*01-DQB1*0501*	0.94	0.020	A	0.47	-0.76
rs34896996	*DQA1*01-DQB1*0503*	0.84	0.030	G	0.18	-1.74
rs62404159	*DQA1*01:03-DQB1*06:03*	0.92	0.005	G	0.36	-1.03
rs17840116	*DQA1*01:02-DQB1*06:09*	0.97	0.010	C	0.38	-0.97
rs117132480	*DQA1*01:03-DQB1*06:01*	0.87	0.060	T	0.57	-0.56
rs28421666	*DQA1*06:01-DQB1*03:01*	0.88	0.060	G	0.63	-0.46
rs142957802	*DQA1*01:02-DQB1*06:01*	0.75	0.020	G	0.47	-0.76
rs112513571	*DQA1*01:02-DQB1*05:02*	0.74	0.050	A	0.31	-1.18

C-GRS, Chinese genetic risk score; OR, odds ratio.

ESM Table 5. Fifteen pairs of haplotypes with interactions are included in the C-GRS.

Haplotype1	Haplotype2	*p*	Beta
*DQA1*03:0X-DQB1*03:03*	*DQA1*01:02-DQB1*06:01*	2.42×10^{-2}	-1.04
*DQA1*03:0X-DQB1*03:03*	*DQA1*01:03-DQB1*06:01*	1.88×10^{-3}	-0.78
*DQA1*03:0X-DQB1*03:03*	*DQA1*06:01-DQB1*03:01*	6.35×10^{-3}	-0.56
*DQA1*03:0X-DQB1*03:03*	*DQA1*05:01-DQB1*02:01*	1.71×10^{-2}	3.71
*DQA1*03:0X-DQB1*03:03*	*DQA1*01:03-DQB1*06:03*	2.19×10^{-2}	2.38
*DQA1*03:0X-DQB1*03:03*	*DQA1*03:03-DQB1*04:01*	1.19×10^{-4}	1.04
*DQA1*01:02-DQB1*06:01*	*DQA1*01:03-DQB1*06:01*	1.88×10^{-3}	0.09
*DQA1*01:02-DQB1*06:01*	*DQA1*06:01-DQB1*03:01*	6.35×10^{-3}	0.19
*DQA1*01:02-DQB1*06:01*	*DQA1*05:01-DQB1*02:01*	9.32×10^{-3}	-1.17
*DQA1*01:03-DQB1*06:01*	*DQA1*06:01-DQB1*03:01*	3.27×10^{-2}	0.60
*DQA1*01:03-DQB1*06:01*	*DQA1*05:05-DQB1*03:01*	5.35×10^{-4}	-0.37
*DQA1*06:01-DQB1*03:01*	*DQA1*05:05-DQB1*03:01*	1.37×10^{-4}	0.21
*DQA1*06:01-DQB1*03:01*	*DQA1*05:01-DQB1*02:01*	4.29×10^{-4}	0.10
*DQA1*05:05-DQB1*03:01*	*DQA1*05:01-DQB1*02:01*	4.23×10^{-4}	-0.29
*DQA1*05:05-DQB1*03:01*	*DQA1*01-DQB1*06:02*	9.59×10^{-3}	-1.52

C-GRS, Chinese genetic risk score.

ESM Table 6. Twelve SNPs from the HLA region distinct from DR-DQ alleles were included in the C-GRS.

CHR	Locus	SNP	BP	Replacing	Beta	Allele	EAF
6	A*2402	rs112944494	29833117	NA	0.54	A	0.211
6	A*0207	rs12174017	29842601	rs9368611	-0.79	T	0.136
6	C*0801	rs2524083	31241737	NA	1.13	T	0.085
6	C*0401	rs112149677	31243313	NA	-0.70	A	0.089
6	B*5401	rs75592100	31328808	NA	0.74	T	0.102
6	B*5801	rs3130063	31558702	NA	1.02	T	0.039
6	*CLIC1*	rs114999198	31703376	NA	1.63	T	0.439
6	*CFB*	rs537160	31916400	NA	-0.29	A	0.045
6	*NOTCH4*	rs8192581	32163664	NA	1.70	T	0.425
6	*TSBP1*	rs3763305	32369488	NA	-0.76	A	0.016
6	Intergenic *DRB9-DQA5*	rs9268833	32428062	NA	1.39	T	0.328
6	Intergenic *MTCO3P1-DQA2*	rs9275599	32682429	NA	0.96	T	0.107

SNP, single nucleotide polymorphism; C-GRS, Chinese genetic risk score; CHR, chromosome; BP, base pair; EAF, effective allele frequency.

ESM Table 7. Eight non-HLA region loci were included in the C-GRS.

CHR	BP	SNP	A1	A2	Gene	*p*	OR	Beta
2	204729153	rs231770	T	C	*CTLA4*	$3.30×10^{-8}$	1.31	0.27
7	34181522	rs10232170	T	C	*BMPER*	$9.90×10^{-9}$	0.68	-0.39
10	6079344	rs11256442	C	T	*IL2RA*	$8.64×10^{-9}$	0.78	-0.25
11	2182224	rs689	T	A	*INS-IGF2*	$5.37×10^{-12}$	2.80	1.03
12	56394954	rs773125	G	A	*SUOX*	$1.26×10^{-8}$	1.35	0.30
12	56435929	rs1131017	G	C	*RPS26*	$4.25×10^{-8}$	0.76	-0.28
12	111856673	rs78894077	T	C	*SH2B3*	$7.58×10^{-11}$	1.83	0.61
12	111933545	rs77465633	A	C	*ATXN2*	$3.91×10^{-12}$	1.93	0.66

C-GRS, Chinese genetic risk score; CHR, chromosome; BP, base pair; SNP, single nucleotide polymorphism; OR, odds ratio.

ESM Table 8. Differentiating T1D vs T2D. Sensitivity and specificity for discriminating between T1D and T2D were assessed across a range of T1D C-GRS cutoff values.

C-GRS cutoff	Sensitivity	Specificity
-0.983	99	21
-0.407	95	45
-0.072	90	61
0.958	65	90
1.211	55	95
2.156	9	99

T1D, type 1 diabetes; T2D, type 2 diabetes; C-GRS, Chinese genetic risk score.

Figures

ESM Figure 1: Flow charts of participants recruited to study.

Discovery cohort:

a

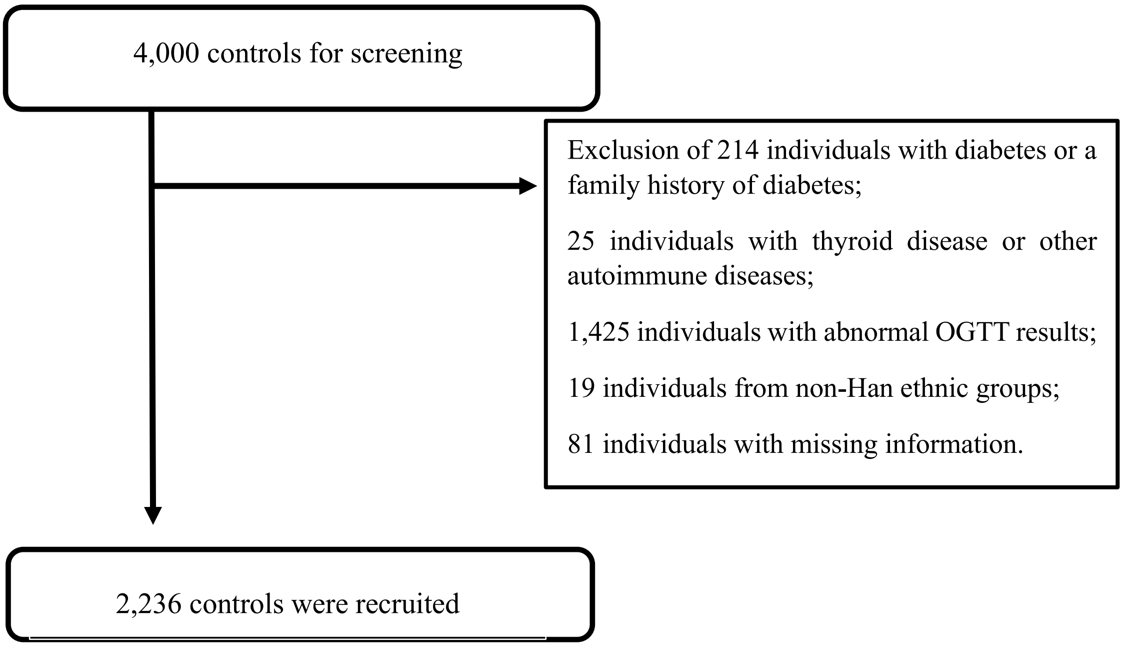

b

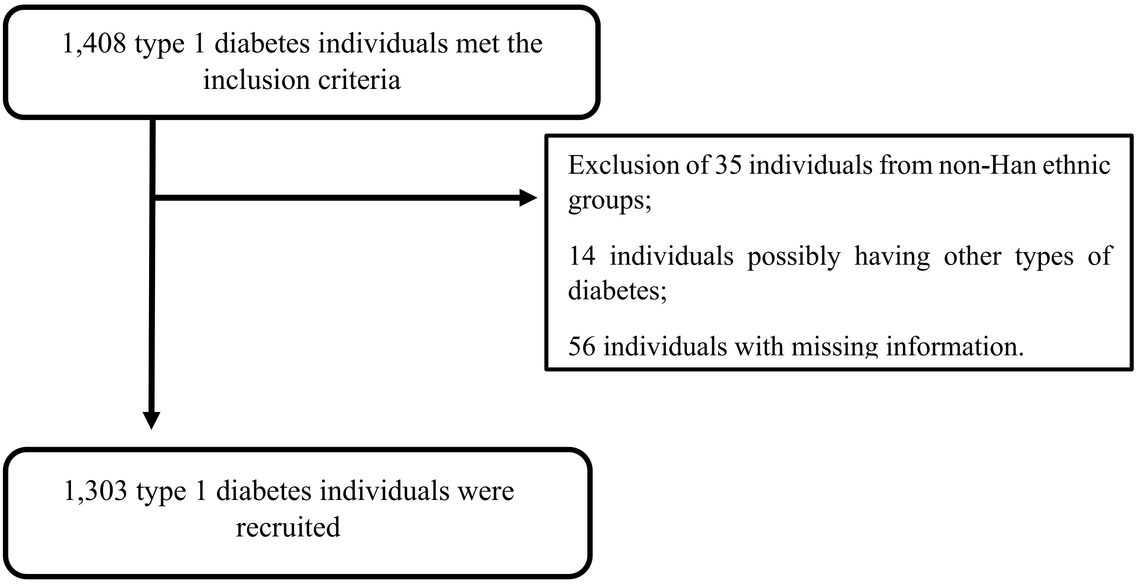

Validation cohort:

c

300 controls for screening

→ Exclusion of 41 individuals with diabetes or a family history of diabetes;

2 individuals with thyroid disease or other autoimmune diseases;

46 individuals with abnormal OGTT results;

0 individual from non-Han ethnic groups;

3 individuals with missing information.

208 controls were recruited

d

270 type 1 diabetes individuals met the inclusion criteria

→ Exclusion of 2 individuals from non-Han ethnic groups;

2 individuals possibly having other types of diabetes;

4 individuals with missing information.

262 type 1 diabetes individuals were recruited

e

1,150 type 2 diabetes individuals met the inclusion criteria

→ Exclusion of 25 individuals from non-Han ethnic groups;

12 individuals having other types of diabetes such as secondary diabetes or gestational diabetes;

33 individuals with missing information.

1,080 type 2 diabetes individuals were recruited

ESM Figure 2**.** Results of a possible newly discovered type 1 diabetes susceptibility locus on near candidate gene *BMPER* on Chromosome 7 in the Chinese population, although it was not strictly replicated. (a) The locuszoom plot displays this locus in the discovery cohort. (b) The forest plot shows the effect size for this locus.

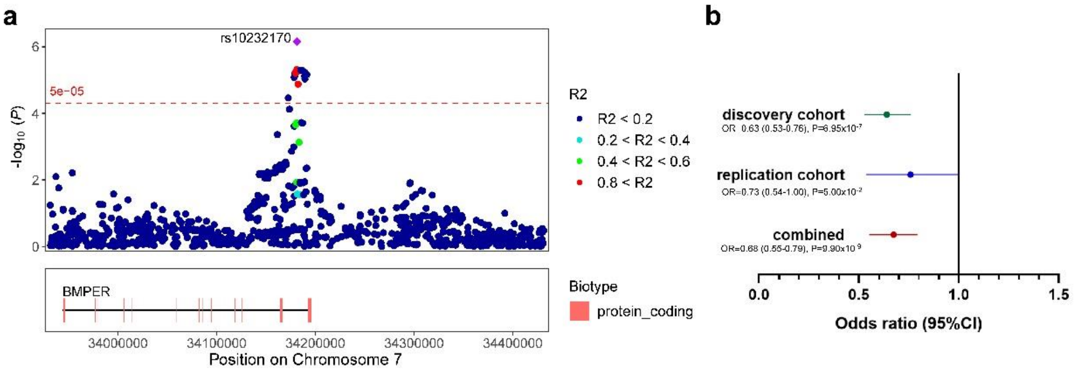

ESM Figure 3**.** GO pathway analysis results from the GWAS of the validated loci. GWAS, genome-wide association study.

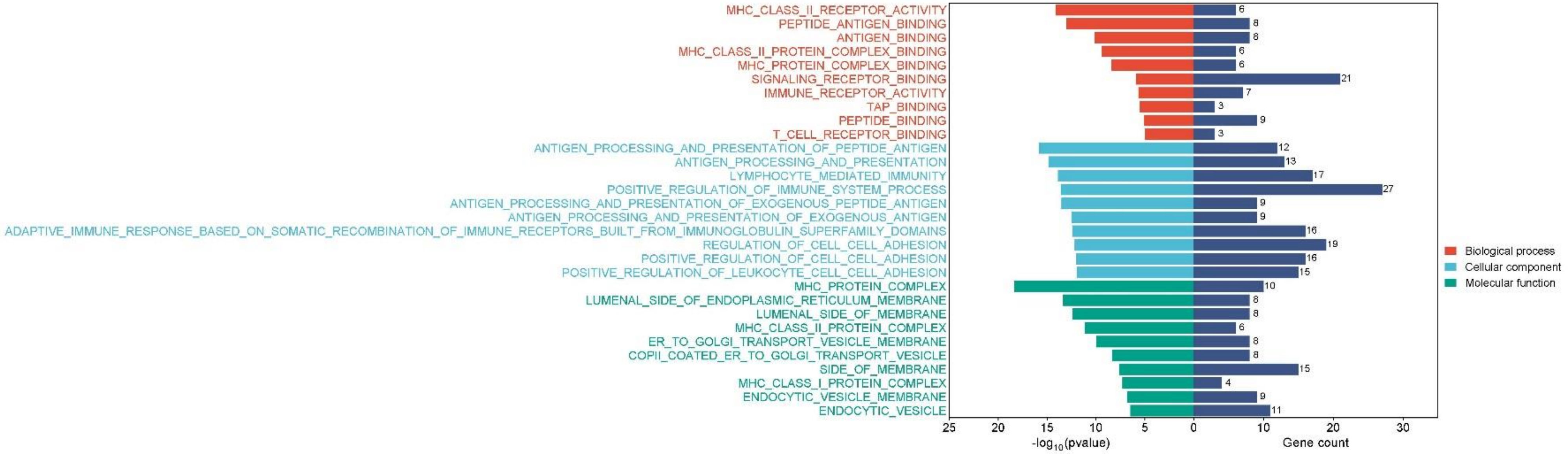

ESM Figure 4. The area under the precision-recall curve was used to generate the AUC for C-GRS in distinguishing type 1 diabetes from controls, based on the discovery cohort. GRS, genetic risk score; AUC, area under the curve.

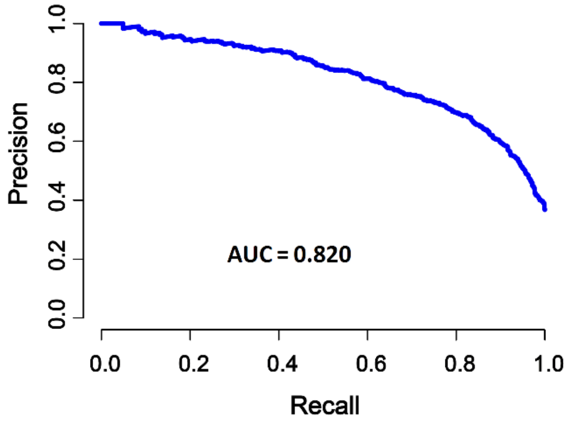

ESM Figure 5. The diagnostic efficacy of C-GRS in stratified analysis of type 1 diabetes by age of onset (discovery cohort). (a) The effectiveness of C-GRS in distinguishing youth-onset type 1 diabetes (age at diagnosis < 20 years) from controls. (b) The effectiveness of C-GRS in distinguishing adult-onset type 1 diabetes (age at diagnosis ≥ 20 years) from controls. GRS, genetic risk score; AUC, area under the curve.

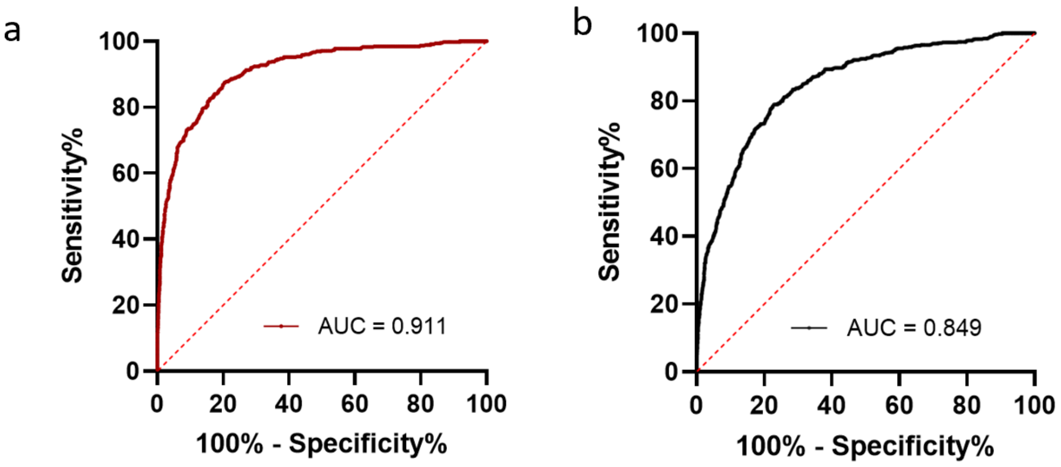

ESM Figure 6**.** Applying type 1 diabetes-GRS2 (established in the populations of European ancestry) to the AUC obtained from the discovery cohort. GRS, genetic risk score; AUC, area under the curve.

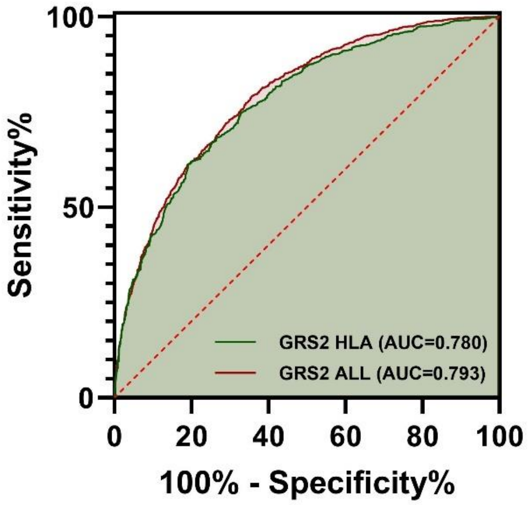

Supplement: Supplementary file 1 — ESM (PDF 671 KB) [file 125_2025_6455_MOESM1_ESM.pdf]
